# Systemic lupus erythematosus and thrombosis

**DOI:** 10.1186/s12959-015-0043-3

**Published:** 2015-04-23

**Authors:** Mario Bazzan, Antonella Vaccarino, Fabio Marletto

**Affiliations:** Haematology and Thrombosis Unit, CMID Department, San Giovanni Bosco Hospital, Piazza Donatore di Sangue 3, 10154 Turin, Italy

**Keywords:** Systemic lupus erythematosus, Thrombosis, Risk factors

## Abstract

Systemic Lupus Erythematosus (SLE) is an acquired, multiorgan, autoimmune disease. Clinical presentation is extremely variable and heterogeneous. It has been shown that SLE itself is an independent risk factor for developing both arterial and venous thrombotic events since SLE patients have an Odds Ratio (OR) for thrombosis that varies depending on the clinical and laboratory characteristics of each study cohort. The risk of developing a thrombotic event is higher in this setting than in the general population and may further increase when associated with other risk factors, or in the presence of inherited or acquired pro-thrombotic abnormalities, or trigger events. In particular, a striking increase in the number of thrombotic events was observed when SLE was associated with antiphospholipid antibodies (aPL). The presence of aPLs has been described in about 50% of SLE patients, while about 20% of antiphospholipid syndrome (APS) patients have SLE. While APS patients (with or without an autoimmune disease) have been widely studied in the last years, fewer studies are available for SLE patients and thrombosis in the absence of APS. Although the available literature undoubtedly shows that SLE patients have a greater prevalence of thrombotic events as compared to healthy subjects, it is difficult to obtain a definite result from these studies because in some cases the study cohort was too small, in others it is due to the varied characteristics of the study population, or because of the different (and very copious) laboratory assays and methods that were used. When an SLE patient develops a thrombotic event, it is of great clinical relevance since it is potentially life-threatening. Moreover, it worsens the quality of life and is a clinical challenge for the clinician.

## Introduction

Systemic Lupus Erythematosus (SLE) is an acquired, multiorgan, autoimmune disease. The clinical presentation is extremely variable and heterogeneous with regard to the possible involvement of various organs and systems, to the varying severity of the clinical picture, and to the abnormalities (when present) of laboratory tests. Diagnosis is often complex, and both clinical and laboratory criteria are usually used. Criteria for the diagnosis of SLE were first published in 1971, while the SLICC (Systemic Lupus International Collaborating Clinics) 2012 criteria are currently used to diagnose SLE (see Table 1) [1-5]. SLE mainly affects women in their childbearing years and may potentially affect any organ or system apparatus. The calculated prevalence is about 20-150/100,000 [6,7], mainly affecting people between 15 and 44 years of age, with a two-fold prevalence for black women [8,9]. On average, Hispanics and African-Americans are diagnosed with SLE at a younger age and with more severe forms of the disease than Caucasians. Cervera [10] showed that 10-year survival in a cohort of 1,000 SLE patients was 90%, and that 25% of the deaths were secondary to active disease, to thrombotic events, or to intercurrent infections. Recently, other authors [11] calculated a 10-year survival rate of 70%*.* According to some authors, thrombotic events and cardiovascular accidents are the first complications of SLE after reactivation (“flares”) of the disease and infections [12]. It has been widely described that SLE itself is an independent risk factor for developing arterial and venous thrombotic events since SLE patients have an Odds Ratio (OR) for thrombosis that varies depending on the clinical and laboratory characteristics of each study cohort. The risk of developing a thrombotic event, which is higher in this setting than in the general population, could further increase when associated with other general, demographic risk factors, or in the presence of inherited or acquired pro-thrombotic abnormalities or of triggering events (such as infections) [13,14]. Thrombotic events are not included in the diagnostic criteria for SLE (see Table 1), but considering that they are a relatively frequent and serious complication of the natural history of the disease, they have been studied in SLE patients both from a physiopathological and from a clinical point of view in an effort to define the therapeutic strategies of prevention and treatment (secondary prevention). In particular, in 1983, a striking increase in thrombotic events was described when the associated presence of anti-phospholipid antibodies (aPL) was observed in SLE patients [15]. The antiphospholipid syndrome (APS) [16] is characterized by the presence of arterial or venous thrombotic events and/or by serious obstetrical complications associated with the persistent presence of aPLs in the serum. “Lupus anticoagulant” (LA) testing identifies the presence of aPLs in the serum that is evaluable by coagulation tests (historically aPTT-based). This name was given since it was initially found in patients with “lupus” and that it prolonged the aPTT, thus simulating the presence of a circulating anticoagulant. The current classification criteria were defined in 2005 at the Consensus Conference of Sydney (see Classification criteria for the APS) [17]. APS can be diagnosed in patients with or without a previously diagnosed autoimmune disease, such as SLE. aPLs have been widely shown to be a significant and independent risk factor for thrombotic events and obstetric complications. It is extremely important to identify the characteristics of aPL positivity according to the Sydney criteria. Three laboratory tests must be performed i.e., LA by functional tests, and anticardiolipin antibodies (ACL) and anti-beta2-GP1 antibodies (anti-β2-GP1) by immunoassay, evaluating both IgG and IgM isotopes. If at least one of the tests is positive [18], it must be confirmed at least 12 weeks after the first assay. A “high titre” of antibodies in the serum, which needs to be confirmed over time, must be present (see classification criteria) to define the positivity of the result. Recently, the term “aPL profile” has been used to define the number and type of positive tests: the higher the number of positive tests, the higher the thrombotic risk. “Triple positivity” (LA plus ACL plus anti-β2-GP1) has the strongest prognostic value in terms of thrombotic events and recurrences [19-21]. Current consensus on classification criteria recommends stratifying the risks faced by APS patients according to their laboratory profile. The overall risk of thrombotic recurrences in APS patients should be stratified considering both clinical and laboratory features. Furthermore, the presence of associated SLE has been considered a factor of “higher risk” for these patients. The presence of aPLs has been described in about 50% of SLE patients, while about 20% of APS patients have SLE [22,23]. Ultimately, when an SLE patient develops a thrombotic event, it is of great clinical relevance since it is potentially life-threatening. Moreover, it worsens the quality of life and is a challenge for the clinician. Herein, we will described some aspects of the association of SLE and thrombosis.

**Table 1:** **SLE diagnostic criteria (modified from** [1]**)**

**Systemic lupus erythematosus: diagnostic criteria** ***(2012 – SLICC)***	
Cutaneous manifestation – 4 Items	Acute Cutaneous Lupus Erythematosus/Subacute Cutaneous Lupus Erythematosus
	Chronic Cutaneous Lupus Erythematosus
	Oral ulcers
	Non-scarring alopecia
Joints – 1 Item	Synovitis > 2 peripheral joints (pain, tenderness, swelling or morning stiffness > 30 min)
Serositis – 1 item	Pleuritis, typical pleurisy ≥ 1 day, history, rub, evidence of pleural effusion, pericarditis, typical pericardial pain ≥ 1 day, EKG evidence of pericardial fusion)
Renal disorder – 1 Item	Urine protein/creatinine ratio or urinary protein concentration of 0.5 g of protein/24 h, Red blood cell casts
Haematological disorder – 3 Items	Haemolytic anaemia
	Leukopenia (<4000/mm3) or lymphopenia (<1000/mm3) separately at least once
	Thrombocytopenia (<100,000/mm3) at least once
Immunologic abnormal – 6 Items	Positive ANA
	Positive anti-dsDNA (except ELISA) on ≥ 2 occasions
	Anti-Sm
	Antiphospholipid antibody (including lupus anticoagulant, false-positive RPR, anti-cardiolipin, anti-beta2glycoprotein1)
	Low complement (C3, C4 or CH50)
	Direct Coombs test in the absence of haemolytic anaemia
Diagnosis	Fulfil 4 items (at least one clinical and one immunologic item)

### Classification criteria for the APS (modified from [17])

#### Clinical criteria

1. **Vascular thrombosis:** one or more clinical episodes of arterial, venous or small vessel thrombosis in any tissue or organ confirmed by imaging or Doppler studies or histopathology (except superficial venous thrombosis, except histopathological evidence of vasculitis)
2. **Pregnancy morbidity:**
  - one or more unexplained deaths of a morphologically normal foetus > 10^th^ week of gestation, or
  - one or more premature births of a morphologically normal neonate < 34^th^ week of gestation because of eclampsia, preeclampsia or placental insufficiency, or
  - three or more unexplained consecutive spontaneous abortions <10^th^ week of gestation with the exclusion of anatomical, hormonal, chromosomal parental abnormalities.

#### Laboratory criteria

1. anticardiolipin antibody IgG and/or IgM isotype in serum or plasma present in medium or high titre (i.e., >40 GPL or MPL or >99^th^ percentile) on 2 or more occasions at least 12 weeks apart
2. lupus anticoagulant present in plasma on 2 or more occasions at least 12 weeks apart
3. anti-beta2 glycoprotein-1 antibody of IgG and/or IgM isotype in serum or plasma, present on 2 or more occasions at least 12 weeks apart

*Definite APS: one clinical criteria and one laboratory criteria*^*c*^*present with first measurement of the laboratory test performed at least 12 weeks from clinical manifestation*^*d*^*.*

^a^coexisting inherited or acquired factors for thrombosis are not reasons for excluding patients from APS trials. Two subgroups of APS patients should be acknowledged according to 1) presence or 2) absence of additional risk factors for thrombosis.

^b^include 1) abnormal or non-reassuring foetal surveillance tests 2) abnormal Doppler flow velocimetry waveform analysis suggestive of foetal hypoxaemia 3) oligohydramnios 4) post natal birth weight less than 10^th^ percentile for gestational age investigators should classify APS patients in I) more than one laboratory criteria present or IIa) ACA present alone or IIb) LA present alone or IIc) anti-beta2GP-I ab present alone^d^ if less than 12 weeks or more than 5 years have gone by since clinical manifestation and confirmation of aPL positivity, then APS should not be defined.

### Search strategy

To identify all available studies, a detailed search pertaining to Systemic Lupus Erythematosus and thrombosis was conducted. A systematic search was performed in the electronic database (PubMed –NCBI) using the following search terms in all possible combinations: systemic lupus erythematosus, arterial thrombosis, vein thrombosis, risk factor, antiphospholipid antibodies, inherited thrombophilia, acquired thrombophilia, cardiovascular disease, atherosclerosis, ethnicity, treatment, antithrombotic treatment, antithrombotic prophylaxis, pregnancy, contraception, catastrophic APS. The last search was performed the 16^th^ January, 2014.

### Physiopathology of atherosclerosis and arterial thrombosis in SLE

Atherosclerosis is a pathological process characterized by the formation of fibro-fatty deposits in the intima layer of large and medium calibre arteries. It is recognized as the most frequent cause of death in Western countries [24]. Studies conducted in the 70's on patients with SLE showed that a bimodal mortality pattern (at 1 year and 8 years after diagnosis) may be observed in patients with SLE. The first peak is due to the disease and to the infectious complications, while the second peak, in the phase of quiescent disease, is due to the long-term glucocorticoid therapy and to cardiovascular morbidity [25]. Over the years there has been a decrease in the number of deaths occurring in the first year after diagnosis, mainly due to the increased effectiveness of the therapies and especially to the prevention of end-stage renal disease, while the mortality for cardiovascular disease (CVD) has not decreased [26] (relative risk compared to the general population for non lethal myocardial infarction RR 10.1, for fatal cardio-coronary heart disease RR 17, for stroke RR 7.9) [27]. A study by the Karolinska Institute [28] on a cohort of SLE patients showed that 50% of SLE patients died of some form of CVD. The main consequences of atherosclerosis in SLE patients include myocardial infarction, stroke and peripheral vascular disease [29]. The presence of atherosclerosis for the stratification of CVD risk was evaluated by Doppler ultrasound which was used to search for and measure carotid plaques and to measure carotid intima media thickness (IMT), while Electron-Beam Computed tomography (EBTC) was used to evaluate the calcium score in the coronary vessels [29]. Results showed early and accelerated onset of atherosclerosis in SLE patients (40% vs. 6-10% of a homogeneous sample among controls) [30,31]. This is associated with the early onset of cardiovascular disease (first event 47–64 years of age) [32], even in pre-menopausal women, thus resulting in a 2- to 10-fold greater risk of developing cardiovascular disease [29] as compared to the general population. There are currently no studies quantifying the burden of early onset atherosclerosis as compared to the development of future cardiovascular events [33]. Atherosclerosis in SLE patients is favoured both by general risk factors and by SLE-related risk factors. General risk factors include age, sex, arterial hypertension, dyslipidemia, obesity, genetic or acquired thrombophilia and ethnicity [34]. The presence of diabetes in SLE patients is surprisingly little-studied: data from a study carried out at Johns Hopkins [35] suggest a two-fold risk of cardiovascular disease. Lastly, the lack of vitamin D [36] in SLE patients has been linked to an increase in atherosclerotic plaques, to high disease activity, to a high body mass index and to the presence of dyslipidemia and insulin resistance. A high homocysteine level is an independent risk factor [37]. The risk factors that are directly related to the pathology (see Table 2) are linked to extensive immune dysregulation, to systemic inflammation, and to endothelial dysfunction (partly mediated by autoantibodies) [29]. SLE patients are known to undergo changes in their lipid profiles, which are mediated by the pro-inflammatory activation of TNF-alpha [29], MCP-1 and IL-6 [38]. Total cholesterol and triglycerides increase, while HDL decreases and loses its anti-inflammatory and scavenger characteristics due to an immune-mediated mechanism. It then becomes pro-inflammatory (piHDL) [39] and correlates to an increased risk of coronary heart disease. It has recently been shown that serum cholesterol efflux capacity (CEC) is impaired in SLE patients thus increasing the atherosclerotic risk of these subjects in a non dependent way with respect to serum HDL levels [40]. Increased lipid oxidation has been reported in these patients [41]. Concomitant nephropathy may contribute to further worsening dyslipidemia. The mechanisms underlying endothelial dysfunction are present from the early stages of the disease with increased expression of cell adhesion molecules (ICAM, VEGF, Von Willebrand factor, VCAM) which are associated with the development of CVD in SLE [32]. The increased expression of Von Willebrand factor (mediated by the production of inflammatory-cytokines) also has a pro-aggregating effect [42]. It has been demonstrated that SLE patients are unable to degrade the NETs (neutrophil extra-cellular traps) complex which regulates apoptotic processes [43]; NETs emerge as a possible mediator of vascular damage and an activator of the thrombotic process. The increase in inflammatory interleukins (IL-17, IL-12 and IL-18) [44], the altered response of B lymphocytes and the production of IgG class autoantibodies with pro-inflammatory meaning [45], and the selective deficiency of T reg lymphocytes are all mechanisms that are present in SLE patients and are associated with an increased risk of CVD [24]. High disease activity is linked to earlier onset of cardiovascular damage, to a worse prognosis (because of the increased risk of CVD), to the increase in IMT thickness (early atherosclerosis) and to the presence of arthritis-serositis (a sign of greater systemic inflammation with increased risk of CVD) [29]. The presence of antibodies to SS-A and SS-B is usually associated with less active disease, but these patients have a pattern of CVD damage and related mortality that is significantly worse [29]. The nephrotic syndrome is correlated with an increased thrombotic risk. In a wide, recently performed meta-analysis on case–control studies of APS patients, APS itself was shown to be related to markers of subclinical atherosclerosis and endothelial damage [46]. In a systematic review on the main predictors of cardiovascular events in SLE patients, [47], the presence of autoantibodies and of neurological disorders were found as “non traditional” risk factors, with an OR of about 5 in both cases. A polymorphism in the toll-like receptor 2 (TLR2) has recently been linked to the pathogenesis of thrombosis in SLE patients. In particular, African Americans and European Americans show an association between TLR2 mutation and thrombosis [48]. Treatment may have an effect on thrombotic risk. Steroids have been reported to increase the atherogenic risk in two ways: the first (direct) via plasma lipoproteins, the second (indirect) by favouring hypertension, diabetes and hyperlipidemia. Moreover, the cumulative dose of steroids, more than the daily dosage alone, seems to be related to the development of atherosclerosis. [49-51]. Antimalarials have an anti-thrombotic, anti-inflammatory effect and control dyslipidemia [52]. Moreover, by blocking toll like receptors 7 and 9, hydroxychloroquine inhibits interferon alpha production which plays a pathogenetic role in SLE pathogenesis [53]. Mycophenolate mofetil reduces the activation of T lymphocytes and increases the presence of regulatory T lymphocytes in carotid plaques [52]. An animal model of SLE showed that Atorvastatin reduces the level of autoantibodies and improves proteinuria and renal histology, however, there is still no general consensus for its extensive use in SLE patients [52]. Treatment with non steroidal anti-inflammatory drugs (NSAIDs) may increase cardiovascular risk (rofecoxib is associated with a greater risk of myocardial infarction, ibuprofen is associated with a higher risk of stroke, diclofenac with higher cardiovascular toxicity, naproxen seems to be the least harmful [54] though it can worsen kidney function. The therapeutic strategy for lowering the risk of CVD should be aimed at an even more aggressive treatment of the disease during the active phase [24]. Other emerging indications include: the immediate cessation of smoking, the use of hydroxychloroquine in all SLE patients who do not have contraindications to such treatment, the use of statins for dyslipidemia, the treatment of arterial hypertension (SBP <120 mmHg), and therapy with acetylsalicylic acid (ASA) which may be useful in SLE patients with general or lupus-related CVD risk factors. The role of immunosuppressive agents in the prevention of atherosclerosis is tentative and must be further investigated [24].

**Table 2:** **Factors influencing atherosclerosis and cardiovascular diseases (CVD) in SLE patients**

**SLE-related factors**	**Effects**
TNF-alpha, MCP-1, IL-6, kidney disease	Dyslipidemia worsening, dysfunctional piHDL production
ICAM, VEGF, vWF, VCAM overexpression	Pro-aggregation/pro-thrombotic behaviour
Acute phase reactants elevation	Tissue damage
INF-alpha elevation	Endothelial damage, pro-thrombotic behaviour
Neutrophil extra-cellular traps	Vascular damage/pro-thrombotic behaviour
IL-17, IL-12, IL-18 elevation, IgG overexpression	Pro-inflammatory
Cystatin C	Early kidney damage marker, pro-inflammatory
SS-A/SS-B positivity profile, photosensibility	Higher risk of CVD
**Medications**	**Effects**
Corticosteroids	Higher risk of CVD
Antimalarial (OH-chloroquine)	Anti-thrombotic, anti-inflammatory, anti-dyslipidemic
Mycophenolate mofetil	Lower immune activity in carotid plaque, protective
HMG-CoA reductase inhibitors (atorvastatin)	Fewer autoantibodies and improvement of kidney function in animal models, to be confirmed in humans
NSAIDs	Higher risk of CVD (less with naproxene)

### Clinical risk factors for thrombosis in SLE: prevention and treatment

#### The clinical risk factors for thrombosis

In SLE have been widely studied. A recent Swedish study [32] showed that in a cohort of 182 SLE patients (with an average follow-up of 8.3 years) who have not had a thrombotic event, 13% developed a cardiovascular event (CVE). CVE events included: ischaemic heart disease (IHD), ischaemic cerebrovascular disease (ICVD), ischaemic peripheral vascular disease (IPVD), or death related to a thrombotic event. The authors concluded that the presence of aPLs increased the Von Willebrand factor, and that the absence of thrombocytopenia significantly correlated to a higher risk of developing an ischaemic event. Retinal vein occlusion is reportedly more frequent in patients with SLE compared to an age-matched control group, with a Hazard Ratio (HR) = 3.883 [55]. Age above 50 years was an independent risk factor, with an HR = 4.8. [55]. A higher incidence of thrombotic events has been reported in SLE patients with lupus nephropathy (LN) [56]. Twenty-five thrombotic events were observed in a cohort of 200 SLE patients who had LN. Sixty-eight % of the events were venous, with an overall incidence of thrombotic events of 29.1 per 1,000 patients/year. The concomitant presence of aPLs (odds ratio 126!), or of sierositis (OR 5), and a history of arterial thrombosis (OR 24) was associated with thrombotic events, while treatment with ACE inhibitors showed a protective effect. Chung et al. evaluated the incidence of venous thromboembolic events in a large Chinese SLE cohort (13,084 patients) and compared it to that of a healthy control group without SLE [57]. After adjusting for age, sex and comorbidities, the risk of developing a deep vein thrombosis (DVT) or a pulmonary embolism (PE) in the SLE patient group was found to be 12.8 and 19.7, respectively, as compared to the control group. The risk of developing IHD, coronary artery disease (CAD) and stroke was calculated in another large cohort of 2,000 patients enrolled in Sweden that included 277 subjects who had American College of Rheumatology (ACR) criteria > 4 [58]. They found an 8- to 9-fold greater risk in middle-aged women with respect to the control population. The presence of IgG ACL was predictive of thrombotic events. A meta-analysis concerning different clinical variables that predict a cardiovascular event in SLE patients was carried out [47]. After a median follow up of 8 years, a prevalence of 25.4% of new thrombotic events was calculated, of which 4% were IHD and 7% were strokes. In this study, the most significant risk factors that were found were male gender, dyslipidemia, family history for CAD, and arterial hypertension, while the presence of autoantibodies and neurological disorders were among the “specific” risk factors. Low correlation was found with respect to organ damage and disease activity. Infectious episodes *per se* could represent an additional risk factor for thrombosis [59,60] in SLE patients. In the last few years, “scores” to calculate the risk of thrombosis in SLE patients have been devised and proposed. Available literature clearly shows that SLE patients have a greater prevalence of thrombotic events with respect to healthy subjects. However, it is difficult to obtain definitive results from these studies because in some cases the study cohort was too small, in other cases it was due to the different characteristics of the study population, or even because of the different (and very copious) laboratory assays and methods that were used.

#### Primary prevention of venous thromboembolism (VTE)

Although SLE *per se* seems to be a risk factor for thrombosis, mainly in the active phases, it is not generally mentioned as a risk factor in the more common risk assessment models (RAM) for medical thromboprophylaxis [61], such as the Caprini RAM [62] or Rogers scores [63]. The presence of LA and of ACL are instead mentioned in these RAM as risk factors. Moreover, the Padua Prediction score risk assessment model [64] also mentions the presence of APS among thrombotic risk factors (regardless of whether it is primary or secondary), thus increasing the score itself. In the absence of available guidelines in this clinical setting, we believe that SLE patients must be considered at higher thrombotic risk, even in the absence of APS and aPLs, mainly during the active phases.

#### VTE and arterial thrombosis treatment

Based on current guidelines, treatment of the acute phase of VTE is no different in these patients from the standard treatment [65]. A higher intensity of anticoagulation therapy (INR 3–4 or low dose ASA associated to avK with an INR range 2–3) (see Table 3) has been recommended in APS patients (with or without SLE) with venous thrombotic recurrence or with arterial events. If indicated, direct oral anticoagulants (dabigatran, rivaroxaban or apixaban) could be used for VTE. Results from studies focusing on this clinical setting are not yet available, though they are in progress. Clinicians must take into consideration that SLE patients often have renal disease and chronic kidney failure, thus there is a need to avoid these drugs or to reduce the doses, according to the manufacturer’s recommendations. The possible association with NSAIDs must also be considered because of the increased bleeding risk. Concerning the optimal duration of anticoagulant treatment, no definite evidence or guidelines are currently available. Considering that a persistent risk of thrombosis is present in these patients, mostly in subjects with SLE and APS, long term anticoagulation is recommended. Shorter anticoagulant treatment should be considered for patients with SLE alone after the first venous thrombotic event, or if SLE is associated with a low risk aPL profile provided that SLE is not in an active phase and that the thrombosis had a well-defined, transitory trigger event. Thrombotic and bleeding risk assessment must be carried out periodically in all of these patients, both at the start of treatment and then at least every year after that. Further evaluation must be performed in case of intercurrent pathologies, or if new drugs to treat SLE are introduced.

**Table 3:** **Treatment recommendations for patients with SLE, associated or not with aPLs or APS, and thrombosis (modified from** [14]**)**

**Clinical scenario**	**Recommendations**
Patients with definite APS (+/− SLE) and first venous event	aVK, INR range of 2.0-3.0
Patients with definite APS (+/− SLE) and venous recurrent thromboembolism during aVK or arterial thrombosis*	aVK, target INR > 3.0^a^
	or
	combination of aspirin (100 mg daily) and aVK, INR range 2.0-3.0^a^
APS patients (without SLE) with a first non cardioembolic cerebral arterial event, low-risk aPL profile, and reversible predisposing factors	Antiplatelet agents
SLE patients with venous or arterial thrombosis who do not fulfil APS laboratory classification criteria	Same as patients from the general population who develop venous or arterial thrombosis

^a^There was a lack of consensus on these recommendations.

APS, antiphospholipid syndrome; INR international normalized ratio; SLE, systemic lupus erythematosus; aPL, antiphospholipid antibodies.

#### Primary prevention of acute arterial diseases

Good quality evidence is also missing in this setting. Some consensus exists [66-69] concerning the use of low dose ASA (i.e., 100 mg daily) in patients with SLE and aPLs who have never had a thrombotic event. Even in the absence of aPLs, associated risk factors such as age, smoking, hypertension, obesity, dyslipidemia, etc. must be taken into consideration when starting primary arterial prevention.

### Pregnancy, contraception and thrombosis

Pregnancy in SLE patients carries a burden of increased risk of morbidity and mortality, both for the mother and for the foetus/newborn [70]. The most frequently reported drawbacks include: pre-eclampsia, pre-term delivery, venous and arterial thrombosis, infections, haematological complications (cytopenias, mainly thrombocytopenia). Increased mortality has been described in this clinical setting [71]. As already reported, a high percentage of SLE patients have aPLs. [70] Furthermore, if these subjects are still asymptomatic (no previous thrombotic event or obstetrical complication), they are at high risk of miscarriage and pregnancy morbidity. In particular, LA positivity [72] proved to have strong positive predictive value for an adverse event during pregnancy. It was recently demonstrated that by using appropriate pharmacological strategies, it is possible to significantly increase the probability of good pregnancy outcome up to 80% of live born babies [70,22]. In SLE patients with asymptomatic aPL positivity, low dose ASA (100 mg/die) is recommended [73]. Low dose ASA associated with LMWH at prophylactic doses (i.e., 4000 U/die) is recommended for SLE with obstetrical APS (miscarriages or foetal loss), but no previous thrombotic event [74,75]. For SLE patients with vascular APS (i.e., a previous thrombotic event), therapeutic LMWH doses (i.e., 100 U/Kg twice a day) are recommended throughout pregnancy and puerperium. Warfarin must be avoided during pregnancy, particularly during the first trimester; data concerning treatment with fondaparinux are scant but encouraging for LMWH-intolerant patients [76]. In the presence of a venous thrombotic event during pregnancy, therapy with full doses of LMWH are recommended, and if possible, factor Xa should be evaluated in order to adjust LMWH dosage. LMWH treatment must be discontinued at least 24 hours before delivery (induction or caesarean section) [77]. LMWH treatment must be extended at least until the 6th week after delivery. Possible further extension of the anticoagulant treatment must be evaluated for each individual patient taking into consideration the presence of aPLs, the aPL profile, the degree of SLE activity, and the degree and quality of the resolution of the thrombotic event. In the clinical context of SLE patients, it is extremely important to provide these patients with information concerning the best contraceptive approach in order to schedule a pregnancy during a stable phase of the disease, and while on drugs that are not contraindicated in pregnancy. Contraceptives containing progesterone alone (progesterone intrauterine devices or drugs) should be considered preferable and safer in SLE patients. Oestro-progestin preparations must be used with great caution and only in SLE patients with stable disease, but are contraindicated in patients with a previous thrombotic event or in the presence of aPLs [78].

### Catastrophic APS

Since a high percentage of patients with SLE are also affected by secondary APS, in this paragraph we will describe a severe and acute thrombotic syndrome that may complicate the outcome of patients affected by APS. Catastrophic APS (CAPS) is a rare and life-threatening form of APS and is characterized by the involvement, over a very short time (less than a week), of several organs/tissues as the target of intravascular thrombosis of microcirculation. The diagnostic criteria for CAPS include: 1) involvement of three or more organs/tissues 2) occurrence of events in less than a week 3) histological evidence of intravascular thrombosis 4) the presence in the patient’s serum/plasma of antiphospholipid antibodies (see Criteria for the classification of catastrophic APS [79]). A previous diagnosis of APS and/or the persistence of clinically significant aPL positivity is useful in the diagnosis of CAPS, however, nearly half of the patients who develop CAPS do not have a history of aPL positivity [80]. CAPS is predominantly characterised by widespread thrombosis of the microcirculation, even though arteries, veins or both may also be involved in the thrombotic process. Histological specimen investigation shows acute thrombotic microangiopathy with inflammatory infiltrate in the interstitial space in one third of cases, immunofluorescence shows strong immunoreactivity and anti-fibrin antibodies, while immune complex deposition is uncommon [81]. An analysis of a large cohort showed that these patients are mainly female (72%) with a mean age of 37 years. Triggering events, such as infections, surgery, discontinuing anticoagulant therapy, medications, obstetric complications or malignancy are usually identifiable [22]. A pre-existing condition of autoimmune disease is quite common (SLE in 40% of patients). CAPS is often accompanied by a systemic inflammatory response syndrome likely due to the extremely extensive tissue damage [82]. Laboratory examinations show positivity in the majority of patients for LA and ACL.

Antinuclear antibodies (ANA) are present in approximately two thirds of patients even though titres are not as high (<1: 320). There may be non-severe thrombocytopenia and haemolytic anaemia (about one third of patients), while the presence of a significant number of schistocytes in the peripheral blood smear is observed in only about one-tenth of patients, probably due to the high speed with which they establish the thrombotic process involving the microcirculation [83]. Differential diagnosis primarily includes haemolytic-uraemic syndrome, thrombotic thrombocytopenic purpura, disseminated intravascular coagulation and heparin-induced thrombocytopenia (HIT). Bleedings and infections often complicate the course of the disease resulting in a worse prognosis. Recent data show a decrease in mortality from 53% to 33% due to a combination of therapeutic strategies in association; these associations include anticoagulants (mainly heparin, which also inhibits complement activation), antiplatelet agents, corticosteroids, plasma-exchange (useful for removing pathological aPLs, cytokines and complement and also incorporating natural anticoagulants like antithrombin and protein C), cyclophosphamide (which could be useful in patients with active autoimmune disease or systemic vasculitis), rituximab (quite promising, especially in patients with severe thrombocytopenia) and high-dose i.v. immunoglobulin (this treatment is based on its ability to block pathological antibodies, to increase clearance, to act on the complement system and to suppress cytokines). Recently, long-lasting remission was obtained in a patient with recurrent CAPS by inhibiting the terminal complement with eculizumab, a recombinant humanized monoclonal IgG2/4 antibody that selectively targets and inhibits the terminal portion of the complement cascade [84]. CAPS patients require adequate management in the intensive care unit which should include haemodialysis, mechanical ventilation or cardiovascular support for shock.

### Criteria for the classification of catastrophic APS (modified from [79])

#### Definite catastrophic APS: All 4 criteria

- Evidence of involvement of three or more organs, systems and/or tissues.^a^
- Development of manifestations simultaneously or in less than a week.
- Confirmation by histopathology of small vessel occlusion in at least one organ or tissue.^b^
- Laboratory confirmation of the presence of antiphospholipid antibodies (lupus anticoagulant and/or anti-cardiolipin antibodies).

#### Probable catastrophic APS:

- All 4 criteria except for the involvement of only two organs, systems and/or tissues
- All 4 criteria except for the absence of laboratory confirmation at least 6 weeks apart due to the early death of a patient never tested for aPLs before catastrophic APS
- 1, 2 and 4
- 1, 3 and 4 and the development of a third event in more than a week but less than a month, despite anticoagulation

^a^Vessel occlusion confirmed by imaging techniques, renal involvement defined as a 50% rise in serum creatinine, severe systemic hypertension (>180/100 mmHg) and/or proteinuria (>500 mg/24 h).

^b^Significant evidence of thrombosis must be present for histopathological confirmation, and vasculitis may coexist.

^c^If not present, previous diagnosis of APS lab confirmation requires the presence of antiphospholipid antibodies which must have been detected on two or more occasions at least 6 weeks ^c^apart (not necessarily at the time of the event), according to the proposed preliminary criteria for the classification of definite APS.

## Conclusions

A great deal of physiopathological data concerning SLE and thrombosis is available. Unfortunately, as far as the clinical approach in this setting is concerned, few guidelines are available and however, are not based on strong evidence. These patients are generally very complicated, and consequently are often treated with an association of multiple drugs. Moreover, SLE patients often have cytopenias, mainly thrombocytopenia, and this complicates the anticoagulant strategies and their standardization. Starting from these premises, the presence of well performed clinical studies in this setting is an unmet clinical need.

## Abbreviations

SLE: Systemic Lupus Erythematosus
OR: Odds Ratio
aPL: Antiphospholipid antibodies
APS: Antiphospholipid syndrome
SLICC: Systemic Lupus International Collaborating Clinics
LA: Lupus anticoagulant
ACL: Anticardiolipin antibodies
anti-β2-GP1: Antibeta 2 GP1 antibodies
CVD: Cardiovascular disease
IMT: Carotid intima media thickness
EBTC: Electron-Beam Computed tomography
NETs: Neutrophil extra-cellular traps
NSAIDs: Non steroidal anti-inflammatory drugs
CVE: Cardiovascular event
IHD: Ischaemic heart disease
ICVD: Ischaemic cerebrovascular disease
IPVD: Ischaemic peripheral vascular disease
HR: Hazard Ratio
LN: Lupus nephropathy
DVT: Deep vein thrombosis
PE: Pulmonary embolism
CAD: Coronary artery disease
VTE: Venous thromboembolism
RAM: Risk assessment models
LMWH: Low molecular weight heparin
CAPS: Catastrophic APS
ANA: Antinuclear antibodies
HIT: Heparin-induced thrombocytopenia

## References

[1] Petri M, Orbai AM, Alarcón GS, Gordon C, Merrill JT, Fortin PR (2012). Derivation and validation of the Systemic Lupus International Collaborating Clinics classification criteria for systemic lupus erythematosus. Arthritis Rheum.

[2] Yu C, Gershwin ME, Chang C (2014). Diagnostic criteria for systemic lupus erythematosus: a critical review. J Autoimmun.

[3] Stephenson JL, Shipman AR (2014). The Systemic Lupus International Collaborating Clinics criteria have replaced the American College of Rheumatology guidelines for the diagnosis of systemic lupus erythematosus. Clin Exp Dermatol.

[4] Fonseca AR, Gaspar-Elsas MI, Land MG, de Oliveira SK (2015). Comparison between three systems of classification criteria in juvenile systemic lupus erythematous. Rheumatology.

[5] Inês L, Silva C, Galindo M, López-Longo FJ, Terroso G, Romão VC, et al. Classification of Systemic lupus erythematosus: Systemic Lupus International Collaborating Clinics versus American College of Rheumatology criteria. Arthritis Care Res 2015 Jan 7. doi: 10.1002/acr.22539. [Epub ahead of print] 10.1002/acr.22539 25581417

[6] Tsokos GC (2011). Systemic Lupus Erythematosus. N Engl J Med.

[7] Rahman A, Isenberg DA (2008). Systemic Lupus Erythematosus N Engl J Med.

[8] Siegel M, Lee SL (1973). The epidemiology of Systemic Lupus Erithematosus. Semin Arthritis Rheum.

[9] Swigris JJ, Fischer A, Gillis J, Gilles J, Meehan RT, Brown KK (2008). Pulmonary and thrombotic manifestations of Systemic Lupus Erythematosus. Chest.

[10] Cervera R, Khamashta MA, Font J, Sebastiani GD, Gil A, Lavilla P (2003). Morbidity and mortality in systemic lupus erythematosus during a 10 year period : a comparison of early and late manifestations in a cohort of 1000 patients. Medicine (Baltimore).

[11] Pons Estel GI, Alarcon GS, Scofield L, Reinlib L, Cooper GS (2010). Understanding the epidemiology and progression of Systemic Lupus Erythematosus. Semin Arthritis Rheum.

[12] Bertoli AM, Fernández M, McGwin G, Alarcón GS, Tan FK, Reveille JD (2006). Systemic lupus erythematosus in a multiethnic US color. XXXIII: clinical (corrected) features, course and outcome in patients with late-onset disease. Arthritis Rheum.

[13] Erkan D (2006). Lupus and thrombosis. J Rheumatol.

[14] Les I, Ruiiz-Irastorza G, Kamashta MA (2012). Intensity and duration of anticoagulant therapy in antiphospholipid syndrome. Sem Thromb Haemost.

[15] Boey ML, Colaco CB, Gharavi AE, Elkon KB, Loizou S, Hughes GR (1983). Thrombosis in systemic lupus erythematosus: striking association with the presence of circulating lupus anticoagulant. Br Med J (Clin Res Ed).

[16] Hughes GRV (1983). Thrombosis, aborption, cerebral disease and the lupus anticoagulant. BMJ.

[17] Miyakis S, Lockshin MD, Atsumi T, Branch DW, Brey RL, Cervera R (2006). International consensus statement on an update of the classification criteria for definite antiphospholipid syndrome (APS). J Thromb Haemost.

[18] Pengo V, Tripodi A, Reber G, Ortel TL, Galli M, De Groot PG (2009). Update of the guidelines for lupus anticoagulant detection. Subcommittee on Lupus Anticoagulant/Antiphospholipid Antibody of the Scientific and Standardisation Committee of the International Society on Thrombosis and Haemostasis. J Thromb Haemost.

[19] Bazzan M, Vaccarino A, Stella S, Bertero MT, Carignola R, Montaruli B (2013). Thrombotic recurrences and bleeding events in APS vascular patients: a review from the literature and a comparison with the APS Piedmont Cohort. Autoimmun Rev.

[20] Bertero MT, Bazzan M, Carignola R, Montaruli B, Silvestro E, Sciascia S (2012). Antiphospholipid syndrome in northwest Italy (APS Piedmont Cohort): demographic features, risk factors, clinical and laboratory profile. Lupus.

[21] Pengo V, Ruffatti A, Legnani C, Gresele P, Barcellona D, Erba N (2010). Clinical course of high-risk patients diagnosed with antiphospholipid syndrome. J Thromb Haemost.

[22] Cervera R, Khamashta MA, Shoenfeld Y, Camps MT, Jacobsen S, Kiss E (2009). Morbidity and mortality in the antiphospholipid syndrome during 5-year period: a multicentre prospective study of 1000 patients. Ann Rheum Dis.

[23] Cervera R, Serrano R, Pons-Estel GJ, Ceberio-Hualde L, Shoenfeld Y, de Ramón E, et al. Morbidity and mortality in the antiphospholipid syndrome during a 10-year period: a multicentre prospective study of 1000 patients. Ann Rheum Dis. 2014 Jan 24doi:10.1136/annrheumdis-2013-204838. [Epub ahead of print]. 10.1136/annrheumdis-2013-204838 24464962

[24] Stojan G, Petri M (2013). Atherosclerosis in systemic lupus erythematosus. J Cardiovasc Pharmacol.

[25] Urowitz MB, Bookman AA, Koehler BE, Gordon DA, Smythe HA, Ogryzlo MA (1976). The bimodal mortality pattern of systemic lupus erythematosus. Am J Med.

[26] Bernatsky S, Boivin JF, Joseph L, Manzi S, Ginzler E, Gladman DD (2006). Mortality in systemic lupus erythematosus. Arthritis Rheum.

[27] Esdaile JM, Abrahamowicz M, Grodzicky T, Li Y, Panaritis C, du Berger R (2001). Traditional Framing-ham risk factors fail to fully account for accelerated atherosclerosis in systemic lupus erythematosus. Arthritis Rheum.

[28] Gustafsson JT, Simard JF, Gunnarsson I, Elvin K, Lundberg IE, Hansson LO (2012). Risk factors for cardiovascular mortality in patients with systemic lupus erythematosus, a prospective cohort study. Arthritis Res Ther.

[29] Gustafsson JT, Svenungsson E (2014). Definitions of and contributions to cardiovascular disease in systemic lupus erythematosus. Autoimmunity.

[30] Roman MJ, Shanker BA, Davis A, Lockshin MD, Sammaritano L, Simantov R (2003). Prevalence and correlates of accelerated atherosclerosis in systemic lupus erythematosus. N Engl J Med.

[31] Asanuma Y, Oeser A, Shintani AK, Turner E, Olsen N, Fazio S (2003). Premature coronary-artery atherosclerosis in systemic lupus erythematosus. N Engl J Med.

[32] Gustafsson J, Gunnarsson I, Börjesson O, Pettersson S, Möller S, Fei GZ (2009). Predictors of the first cardiovascular event in patients with systemic lupus erythematosus – a prospective cohort study. Arthritis Res Ther.

[33] Hillen T, Nieczaj R, Münzberg H, Schaub R, Borchelt M, Steinhagen-Thiessen E (2000). Carotid atherosclerosis, vascular risk profile and mortality in a population-based sample of functionally healthy elderly subjects: the Berlin ageing study. J Intern Med.

[34] Manzi S, Meilahn EN, Rairie JE, Conte CG, Medsger TA, Jansen-McWilliams L (2007). Age-specific incidence rates of myocardial infarction and angina in women with systemic lupus erythematosus: comparison with the Framingham Study. Am J Epidemiol.

[35] Magder LS, Petri M (2012). Incidence of and risk factors for adverse cardiovascular events among patients with systemic lupus erythematosus. Am J Epidemiol.

[36] Ravenell RL, Kamen DL, Spence JD, Hollis BW, Fleury TJ, Janech MG (2012). Premature atherosclerosis is associated with hypovitaminosis D and angiotensin-converting enzyme inhibitor non-use in lupus patients. Am J Med Sci.

[37] Roman MJ, Crow MK, Lockshin MD, Devereux RB, Paget SA, Sammaritano L (2007). Rate and determinants of progression of atherosclerosis in systemic lupus erythematosus. Arthritis Rheum.

[38] Urowitz MB, Gladman DD, Tom BD, Ibañez D, Farewell VT (2008). Changing patterns in mortality and disease outcomes for patients with systemic lupus erythematosus. J Rheumatol.

[39] McMahon M, Grossman J, Skaggs B, Fitzgerald J, Sahakian L, Ragavendra N (2009). Dysfunctional proinflammatory high-density lipoproteins confer increased risk of atherosclerosis in women with systemic lupus erythematosus. Arthritis Rheum.

[40] Ronda N, Favari E, Borghi MO, Ingegnoli F, Gerosa M, Chighizola C (2014). Impaied serum Cholesterol efflux capacity in rheumatoid arthritis and sistemi lupus erythematosus. Ann Reum Dis.

[41] Frostegård J, Svenungsson E, Wu R, Gunnarsson I, Lundberg IE, Klareskog L (2005). Lipid peroxidation is enhanced in patients with systemic lupus erythematosus and is associated with arterial and renal disease manifestations. Arthritis Rheum.

[42] Spiel AO, Gilbert JC, Jilma B (2008). Von Willebrand factor in cardiovascular disease: focus on acute coronary syndromes. Circulation.

[43] Knight JS, Carmona-Rivera C, Kaplan M (2012). Proteins derived from neutrophil extracellular traps may serve as self-antigens and mediate organ damage in autoimmune diseases. Front Immunol.

[44] Lahoute C, Herbin O, Mallat Z, Tedgui A (2011). Adaptive immunity in atherosclerosis: mechanisms and future therapeutic targets. Nat Rev Cardiol.

[45] Caligiuri G, Nicoletti A, Poirier B, Hansson GK (2002). Protective immunity against atherosclerosis carried by B cells of hypercholesterolemic mice. J Clin Invest.

[46] Ambrosino P, Lupoli R, DiMinno A, Lervolino S, Peluso MND DiMinno R (2014). Markers of gardiovascular risk in patients with antiphospholipid syndrome: meta-analysis of literatute studies. Ann Med.

[47] Ballocca F, D'Ascenzo F, Moretti C, Omedè P, Cerrato E, Barbero U, Abbate A, Bertero MT, Zoccai GB, Gaita F : Predictors of cardiovascular events in patients with systemic lupus erythematosus (SLE): a systematic review and meta-analysis. Eur J Prev Cardiol. 2014; [Epub ahead of print] 10.1177/2047487314546826 25139772

[48] Kaiser G, R Tang LF, Taylor KE, Sterba K, Nititham J, Brown EE (2014). A polymorphism in TLR2 is associated with arterial thrombosis in a multiethnic population of patients with systemic lupus erythematosus. Arthritis. Rheumatol.

[49] Bruce IN, 44 (2005). “Not only….but also”: factors that contribute to accelerated atherosclerosis and premature coronary heart disease in systemic lupus erythematosus. Rheumatology.

[50] Gorman C, Isemberg D (2004). Atherosclerosis in lupus. Rheumatology.

[51] Doria A, Shoenfeld Y, Wu R, Gambari PF, Puato M, Ghirardello A (2003). Risk factors for subclinical atherosclerosis in a prospective cohort of patients with systemic lupus erythematosus. Ann Rheum Dis.

[52] Tazi Mezalek Z, Harmouche H, Ammouri W, Maamar M, Adnaoui M, Cacoub P (2014). Atherosclerosis in systemic lupus erythematosus. Presse Med.

[53] Costedoat-Chalumeau N, Dunoguè B, Morel N, Le Guern V, Guettrot-Imbert G (2014). Hydroxychloroquine: a multifaceted treatment in lupus. Presse Med.

[54] Knight JS, Kaplan MJ (2013). Cardiovascular disease in lupus: insights and updates. Curr Opin Rheumatol.

[55] Trelle S, Reichenbach S, Wandel S, Hildebrand P, Tschannen B, Villiger PM (2011). Cardiovascular safety of non-steroidal anti-inflammatory drugs: network meta-analysis. BMJ.

[56] Yen YC, Weng SF, Chen HA, Lin YS (2013). Risk of retinal vein occlusion in patients with systemic lupus erythematosus: a population-based cohort study. Br J Ophthalmol.

[57] Zaldívar-alcántara H, Herrera-jiménez LE, Dehesa-lópez E, Correa-rotter R (2013). Risk factors for the development of thrombotic complication in patients with lupus erythematosus and lupus nephropatic. Rev Invest Clin.

[58] Chung WS, Lin CL, Chang SN, Lu CC, Kao CH (2014). Systemic lupus erythematosus increases the risks of deep vein thrombosis and pulmonary embolism: a nationwide cohort study. J Thromb Haemost.

[59] Bengtsson C, Ohman ML, Nived O, Rantapää Dahlqvist S (2012). Cardiovascular event in systemic lupus erythematosus in northern Sweden: incidence and predictors in a 7-year follow-up study. Lupus.

[60] Baronaite Hansen R, Jacobsen S (2014). Infections Increase Risk of Arterial and Venous Thromboses in Danish Patients with Systemic Lupus Erythematosus: 5102 Patient-years of Followup. J Rheumatol.

[61] Kahn SR, Lim W, Dunn AS, Cushman M, Dentali F, Akl EA (2012). Prevention of VTE in Nonsurgical patients. CHEST.

[62] Caprini JA (2010). Risk assessment as a guide for the prevention of the many faces of venous thromboembolism. Am J Surg.

[63] Rogers SO, Kilaru RK, Hosokawa P, Henderson WG, Zinner MJ, Khuri SF (2007). Multivariable predictors of postoperative venous thromboembolic events after general and vascular surgery: results from the patient safety in surgery study. J Am Coll Surg.

[64] Barbar S, Noventa F, Rossetto V, Ferrari A, Brandolin B, Perlati M (2010). A risk assessment model for the identification of hospitalized medical patients at risk for venous thromboembolism: the Padua Prediction Score. J Thromb Haemost.

[65] Kearon C, Akl EA, Comerota AJ, Prandoni P, Bounameaux H, Goldhaber SZ (2012). Antithrombotic therapy for VTE disease. CHEST.

[66] Bazzan M, Vaccarino A (2009). Aspirin in asymptomatic patients with confirmed positivity of antiphospholipid antibodies: only in selected, high risk patients. Intern Emerg Med.

[67] Metjian A, Lim W. ASH evidence-based guidelines: should asymptomatic patients with antiphospholipid antibodies receive primary prophylaxis to prevent thrombosis? Hematology Am Soc Hematol Educ Program. 2009;247–9. 10.1182/asheducation-2009.1.247 20008205

[68] Arnaud L, Mathian A, Ruffatti A, Erkan D, Tektonidou M, Cervera R (2014). Efficacy of aspirin for the primary prevention of thrombosis in patients with antiphospholipid antibodies: an international and collaborative meta-analysis. Autoimmun Rev.

[69] Bertero MT (2012). Primary prevention in antiphospholipid antibody carriers. Lupus.

[70] Lateef A, Petri M : Managing lupus patients during pregnancy. Best Pract Res Clin Rheumatol. 2013 10.1016/j.berh.2013.07.005 PMC3834352 24238698

[71] Clowse ME, Jamison M, Myers E, James AH (2008). A National study of the complication of lupus in pregnancy. Am J Obstet Gynecol.

[72] Lockshin MD, Kim M, Laskin CA, Guerra M, Branch DW, Merrill J (2012). Prediction of adverse pregnancy outcome by the presence of lupus anticoagulant, but not anticardiolipin antibody, in patients with antiphospholipid antibodies. Arthritis Rheum.

[73] Del Ros T, Ruffatti A, Visentin MS, Tonello M, Calligaro A, Favaro M (2013). Treatment of 139 pregnancies in antiphospholipid positive women not fullifilling criteria for antiphospholipid syndrome: a retrospective study. J Rheumatol.

[74] Empson M, Lassere M, Craig J, Scott J: Prevention of recurrent miscarriage for women with antiphospholipid antibody or lupus anticoagulant. Cochrane Database Syst Rev 2005 10.1002/14651858.CD002859.pub2 PMC6768987 15846641

[75] Mak A, Cheung MW, Cheak AA, Ho RC (2010). Combination of heparin and aspirin is superior to aspirin alone in enhancing live births in patient with recurrent pregnancy loss and positive antiphospholipid antibodies: a meta analysis of randomized controlled trials and meta regression. Rheumatology.

[76] Giannubilo SR, Tranquilli AL (2012). Anticoagulant therapy during pregnancy for maternal and fetal acquired and inherited thrombofilia. Curr Med Chem.

[77] Bates SM, Greer IA, Middeldorp S, Veenstra DL, Prabulos AM, Vandvik PO (2012). VTE, thrombophilia, antithrombotic therapy, and pregnancy: Antithrombotic Therapy and Prevention of Thrombosis, 9th ed: American College of Chest Physicians Evidence-Based Clinical Practice Guidelines. American College of Chest Physicians CHEST.

[78] Sammaritano L (2014). Contraception in patients with sistemic lupus erythematosus and antiphospholIpid syndrome. Lupus.

[79] Nayer A, Ortega LM (2014). Catastrophic antiphospholipid syndrome: a critical review. J Nephropathol.

[80] Asherson RA, Espinosa G, Cervera R, Font J, Reverter JC (2002). Catastrophic antiphospholipid syndrome: proposed guidelines for diagnosis and treatment. J Clin Rheumatol.

[81] Tektonidou MG, Sotsiou F, Moutsopoulos HM (2008). Antiphospholipid syndrome (APS) nephropathy in catastrophic, primary, and systemic lupus erythematosus-related APS. J Rheumatol.

[82] Rangel ML, Alghamdi I, Contreras G, Harrington T, Thomas DB, Barisoni L (2013). Catastrophic Antiphospholipid Syndrome with Concurrent Thrombotic and Hemorrhagic Manifestations. Lupus.

[83] Sciascia S, Lopez-Pedrera C, Roccatello D, Cuadrado M (2012). Catastrophic antiphospholipid syndrome (CAPS). Best Pract Res Clin Rheumatol.

[84] Bucciarelli S, Espinosa G, Cervera R, Erkan D, Gómez-Puerta JA, Ramos-Casals M (2006). European Forum on Antiphospholipid Antibodies. Mortality in the catastrophic antiphospholipid syndrome: causes of death and prognostic factors in a series of 250 patients. Arthritis Rheum.

